# Recent Progress of Deubiquitinating Enzymes in Human and Plant Pathogenic Fungi

**DOI:** 10.3390/biom12101424

**Published:** 2022-10-04

**Authors:** Weixiang Wang, Xuan Cai, Xiao-Lin Chen

**Affiliations:** 1 Beijing Key Laboratory of New Technology in Agricultural Application, National Demonstration Center for Experimental Plant Production Education, College of Plant Science and Technology, Beijing University of Agriculture, Beijing 102206, China; 2 State Key Laboratory of Agricultural Microbiology and Provincial Hubei Key Laboratory of Plant Pathology, College of Plant Science and Technology, Huazhong Agricultural University, Wuhan 430070, China

**Keywords:** deubiquitination, deubiquitinating enzymes, fungal virulence, pathogenic fungi, infection process

## Abstract

In eukaryotic cells, a large number of proteins are modified by ubiquitination, which leads to proteasomal degradation or change in protein function. The protein ubiquitination process can be reversed by a process called deubiquitination, which plays an important regulatory mechanism in cellular control. Deubiquitination is catalyzed by deubiquitinating enzymes (DUBs); the cysteine proteases specifically cleave off ubiquitin from ubiquitinated substrates or ubiquitin precursors. Over the past two decades, components of different DUB families have been found to play important roles in both human and plant pathogenic fungi. Given the importance of DUBs for fungal development and virulence, in this review, we concentrate on recent findings and new insights into the roles of DUBs in different fungal pathogens, with a focus on infection-related morphogenesis and virulence, as well as their roles in development and stress response. We also summarize the DUBs-mediated regulatory mechanisms during the above processes. These findings should allow us to develop novel inhibitors to control fungal pathogens.

## 1. Introduction

Ubiquitination is one of the most important post-translational modifications that binds ubiquitin proteins to target proteins [1]. This modification process is catalyzed sequentially by the action of three hierarchical enzymes, i.e., a ubiquitin-activating enzyme (E1), a ubiquitin-conjugating enzyme (E2), and a ubiquitin ligase (E3). During this cascade, there are hundreds of E3 ligases with different families for selective modification of the target proteins. Then, the carboxyl group of ubiquitin glycine and the ε-amino group of the substrate form an isopeptide bond [2]. There are seven internal lysine residues on ubiquitin that can be attached to target proteins, including K6, K11, K27, K29, K33, K48, and K63. A target protein can be attached with either a mono- or poly-ubiquitin chain [2]. The K48-linked ubiquitin chain attached on target proteins is the most common linkage type, which acts as a proteolysis marker, and leads to protein degradation by the proteasome system [3]. Other atypical ubiquitin chains that are attached on target proteins are usually involved in regulating protein localization, protein–protein interactions, and protein/enzyme activity [3]. Some other proteins, the so-called ubiquitin-like modifiers (UBLs), such as NEDD8, SUMO, and ISG15, can also modify target proteins. UBLs are also attached to target proteins by E1-E2-E3 enzyme cascades [4]. Neddylation and sumoylation are two commonly found ubiquitin-like modifications in eukaryotic cells [4].

**Figure 1:**
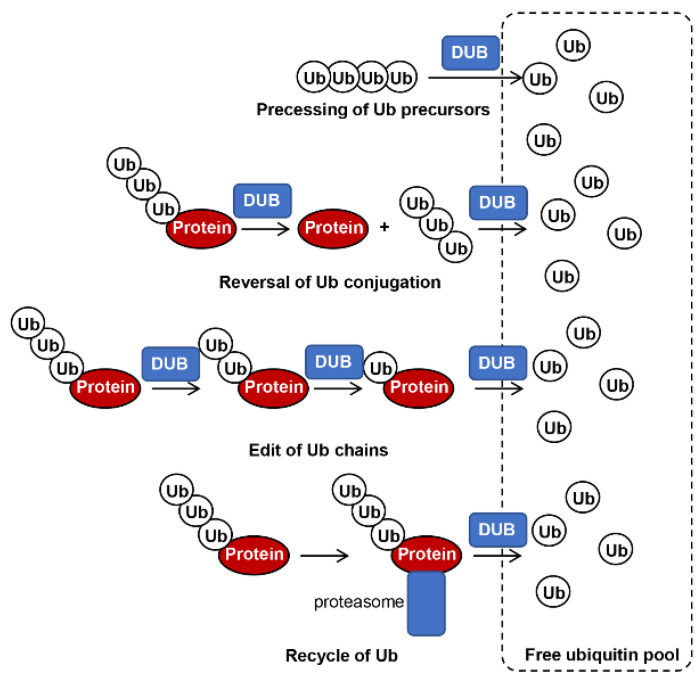
General roles of DUBs in fungi. DUBs play different roles in processing of ubiquitin precursors, reversal of ubiquitin conjugation, editing of ubiquitin chains, and recycling of ubiquitin from the 26 S proteasome, which all facilitate ubiquitin re-entry to the free ubiquitin pool.

Ubiquitination and ubiquitin-like modifications are reversible modifications that can be reversed by the deubiquitination process. A large number of deubiquitinating enzymes (DUBs) are used by the cells to catalyze the deubiquitination process [5]. During this process, DUBs are used to cleave ubiquitin molecules from the substrates, therefore, generating a ubiquitin pool for recycling of ubiquitin (Figure 1) [6]. Similarly, UBL-related DUBs, such as SUMO-specific protease (SENP) and NEDD8-specific protease (NEDP), respectively, cleave SUMO and NEDD8 forms target proteins or UBL precursors [7].

There are around 100 DUBs in humans that belong to five families, including ubiquitin-specific proteases (USPs), ubiquitin C-terminal hydrolases (UCHs), ovarian tumor proteases (OTUs), JAB1/MPN/MOV34 metalloproteases (JAMMs), Machado–Josephin domain containing proteases (MJDs), and motif interacting with Ub-containing novel DUB family (MINDY) [8,9]. To date, the structures, functions, and mechanisms of the DUBs have been revealed by some studies, and well summarized in human diseases such as cancer, neurodegenerative diseases, brain diseases, as well as immunity [10,11,12,13].

Fine-tuning of the development, stress response, nutrient utilization, and infection processes requires a balance between the addition and removal of ubiquitin moieties in both human and plant pathogenic fungi. In this review, we introduce the basic knowledge of DUBs and summarize the roles and mechanisms of DUBs in control of the development, stress response, nutrient utilization, and infection processes in human and plant pathogenic fungi. In particular, we highlight the advances in mechanisms of fungal DUBs related to pathogenesis. The information in this review extends the body of knowledge regarding the roles that DUBs play in regulating fungal infection, and we also describe how targeting DUBs could be utilized to develop DUB inhibitors that could be applied to yield therapies for fungal diseases, both in humans and plants.

## 2. Summary of DUB Studies in Different Human and Plant Pathogenic Fungi

There are around 93 DUBs that have been identified in humans, which have been classified into five families, including 54 USPs/UBPs (ubiquitin-specific proteases), 16 OTUs (ovarian tumor proteases), 4 UCHs (ubiquitin C-terminal hydrolases), 4 MJDs (Machado–Joseph deubiquitinases), and 7 JAMMs (JAB1/MPN/MOV34 metalloproteases) [9]. These subclasses of DUBs are different in their catalytic domains and other domains. We used the EnsemblFungi genome database (http://fungi.ensembl.org/index.html (accessed on 15 September 2022)) to retrieve all putative DUBs from the genomes of yeast *Saccharomyces cerevisiae*, human fungal pathogen *Cryptococcus neoformans,* and plant fungal pathogen *Magnaporthe oryzae* by selecting genes encoding proteins containing Ub protease domains. We identified the five known families of DUBs. This analysis indicated that the *S. cerevisiae* encodes approximately 22 putative DUBs (16 UBPs (ubiquitin-specific protease domain family), 1 UCH, and 2 MINDYs (MIU-containing novel DUB family), 2 OTUs, and 1 JAMM); *C. neoformans* encodes approximately 16 putative DUBs (11 UBPs, 1 UCH, 1 OTU, and 3 JAMMs); and *M. oryzae* encodes approximately 20 putative DUBs (11 UBPs, 2 UCHs, 1 MINDYs, 2 OTUs, and 4 JAMMs) (Table 1).

**Figure 2:**
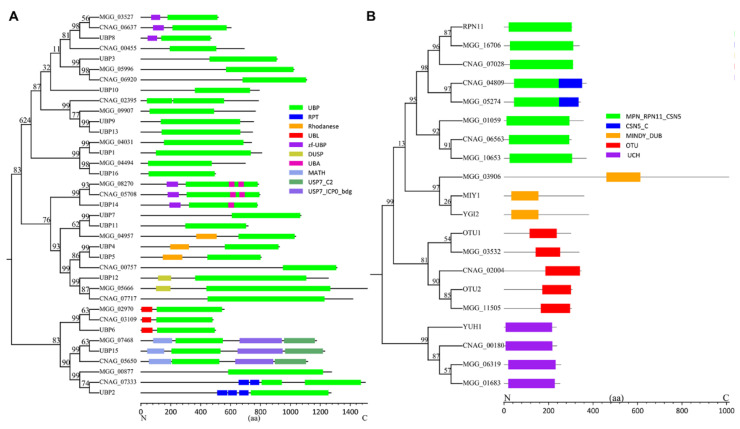
Phylogenetic tree and domain structure of DUBs in *S. cerevisiae*, *C. neoformans,* and *M. oryzae*: (**A**) Phylogenetic tree and domain structures of ubiquitin-specific proteases (UBPs) (zf-UBP, Zn-finger in ubiquitin-hydrolases and other protein; UBA, ubiquitin associated domain; RPT, repeated domain in UCH-protein; UBL, ubiquitin-like domain; DUSP, domain present in ubiquitin-specific protease; MATH, meprin and TRAF homology; USP7_C2, ubiquitin-specific protease C-terminal; USP7_ICP0_bdg, ICP0-binding domain of ubiquitin-specific protease 7); (**B**) phylogenetic tree and domain structures of UCHs, OTUs, MINDY, and JAMM family proteins. The domain architectures of DUBs are drawn by referring to predicted results from databases of Pfam, PROSITE, SMART, and Interpro.

The UBP (USPs in human) family represents the largest DUBs in fungal genomes that mainly process larger leaving groups. The catalytic domain of UBPs is well-conserved in this family, but distinct from other domains, including domains of Zn-finger in ubiquitin-hydrolases and other protein (zf-UBP), ubiquitin-associated domain (UBA), repeated domain in UCH-protein (RPT), rhodanese, ubiquitin-like domain (UBL), domain present in ubiquitin-specific protease (DUSP), meprin and TRAF homology (MATH), ubiquitin-specific protease C-terminal (USP7_C2), and ICP0-binding domain of ubiquitin-specific protease 7 (USP7_ICP0_bdg) (Figure 2A). Regarding *S. cerevisiae*, UBP5 contains a rhodanese domain, UBP12 contains a DUSP domain, UBP6 contains a UBL domain, and UBP2 contains several RPT domains. UBP8 and UBP14 contain a zf-UBP domain, and the latter also contains a UBA domain in the UBP domain region. UBP15 contains a MATH domain, a USP7_C2 domain, and a USP7_ICP0_bdg domain. There are no predicted other known domains in UBP3, UBP10, UBP9, UBP13, UBP1, and UBP16. These domains may function as regulatory domains to facilitate different roles of UBPs.

The UCH family of DUBs consists of only one protein (YUH1) in yeasts *S. cerevisiae*, and *C. neoformans* (CNAG_00180), but contains two proteins (MGG_06319 and MGG_01683) in *M. oryzae* (Figure 2B). Studies have suggested that the UCHs prefer to cleave small protein substrates from ubiquitin, and recycle ubiquitin inappropriately conjugated to substrates (Amerik and Hochstrasser, 2004). UCHs may also be involved in processing newly synthesized ubiquitin precursors, while the specific functions of UCHs remain largely unknown. The OTU family of DUBs consists of two proteins (OTU1 and OTU2) in *S. cerevisiae*, one protein in *C. neoformans* (CNAG_02004), and two proteins (MGG_03532 and MGG_11505) in *M. oryzae*. The OTU family of DUBs is defined on the basis of their homology to the ovarian tumor gene, which functions in the development of the ovaries in fruit flies [14]. The JAMM family of DUBs contains one protein (RPN11) in *S. cerevisiae*, three proteins in *C. neoformans* (CNAG_07028, CNAG_04809, and CNAG_06563), and four proteins (MGG_16706, MGG_05274, MGG_01059, and MGG_10653) in *M. oryzae* (Figure 2B). The CSN5 protein homologs contain a metalloprotease motif (JAMM) in the MPN domain and a CSN5_C domain, making them the catalytic center for the COP9 signalosome complex (CSN complex) [15]. The CSN complex functions as a deneddylation DUB to remove the UBL NEDD8 from cullin proteins [16]. Interestingly, in *S. cerevisiae*, no CSN5 homologue is identified, but in *C. neoformans* and *M. oryzae*, they have one homologue (CNAG_04809 and MGG_05274), suggesting an evolutionary difference in them. The MINDY family of DUBs contains two proteins (MIY1 and YGI2) in *S. cerevisiae*, one protein in *M. oryzae* (MGG_03906), but is absent in *C. neoformans*.

Some other predicted DUBs could also function toward moieties of UBLs, such as NEDD8, SUMO, ISG15, and ATG8 [4]. For example, in *S. cerevisiae*, UBL ATG8 can be processed by the protease ATG4. Likewise, in *S. cerevisiae*, UBL Smt3 (SUMO) can be processed by the cysteine proteases Ulp1 and Ulp2.

## 3. Biological Functions of DUBs in Different Pathogenic Fungi

### 3.1. Overview of Roles of Ubiquitination and DUBs in Different Pathogenic Fungi

The functions of ubiquitination or ubiquitin-like modification have been widely revealed in both human fungal pathogens (*C. albicans* and *C. neoformans*) and plant fungal pathogens (*M. oryzae*, *Fusarium oxysporum, and Cryphonectria parasitica*) [17,18,19,20,21,22,23,24,25,26,27]. In fungal pathogens, the role of ubiquitination has been studied by analyzing ubiquitin-related genes, and it was found that ubiquitination played important roles in fungal development, stress resistance, nutrient utilization, and fungal virulence [19,20,21,22,25,27]. For example, in *C. albicans*, deletion of the ubiquitin gene *UBI4* affects the cell cycle, morphology, resistance to thermal stress, oxidative stress, and cell wall perturbing stresses, and results in catabolism block and virulence reduction [19,20,21]. In *C. neoformans*, deletion of the ubiquitin gene *UBI1* leads to defects in vegetative growth, reduction in melanin synthesis, and attenuation in virulence during infection [22]. Likewise, in the plant pathogen *M. oryzae*, *UBI4* deletion also results in defects of growth and loss of virulence, and it also leads to significant reductions in sporulation, spore germination, as well as appressorium formation [25]. The ubiquitin gene *UBI4* has also been found to be important for fungal development, stress response, and virulence in other plant pathogenic fungi, such as *Cryphonectria parasitica* [27].

Genes encoding E2 ubiquitin-conjugating enzymes and E3 ubiquitin ligases have also been studied in human fungal pathogens and plant fungal pathogens. Explaining the dynamics of E2-E3 profiling may help to decode which E2:E3 pairs are true partners. In humans, it is very important to determine specific E2-E3 pairing in different disease states, because an accurate description of E2-E3 is crucial in the development of therapeutic drugs. Future research on E2-E3 coordination selectivity will contribute to early diagnosis and the development of cancer therapeutics [28]. Disruption of these E2 and E3 encoding genes also affects fungal development, stress response, and virulence. *C. albicans* Rad6 is a ubiquitin conjugating enzyme, which positively regulates UV damage repair but negatively regulates hyphal growth [29]. While, in *M. oryzae*, Rad6 plays key roles in development and infection of plant hosts [30]. It has been widely reported that homologues of F-box proteins were associated with virulence of important fungal pathogens [31], including GRR1 in *C. albicans* [32], GRR1 in *Gibberella zeae* [33], CDC4 in *M. oryzae* [34], and *C. albicans* [35,36]. For example, in *C. neoformans*, the GRR1 homolog FBP1 is essential for mycelial growth, sexual reproduction, and virulence [24,37]. In *M. oryzae*, two homologs of GRR1 are required for fungal pathogenicity to rice [34]. The roles of subunits of the SCF complex (E3 ligase component) in pathogenic fungi, such as cullin CDC53 in *C. albicans* [38], FBX15 in *A. fumigatus* [39], FRP1 in *F. oxysporum* [40], MoFWD1, MoCDC4, and MoFBX15 in *M. oryzae* [34] have also been reported. In *C. albicans*, another SCF complex component CDC4 is also essential for morphogenesis and biofilm formation [35,36]. F-box proteins also play key roles in human cancer initiation and progression, such as FBXL8 and FBXO43 [41,42]. Therefore, the roles of ubiquitination revealed by ubiquitin encoding genes and E2/E3 encoding genes in both human and plant pathogenic fungi demonstrate key roles in various biological functions.

Although the function of ubiquitination has been well revealed [17,18], deubiquitination mediated by DUBs has not been well summarized in pathogenic fungi. On the one hand, similar to ubiquitination, the biological functions of DUBs are quite different. On the other hand, DUBs are highly redundant in function, and it is hard to specify physiological functions to each of the DUBs. A comparative investigation of the single DUB gene to determine phenotypic differences is required. Several studies have addressed this issue in yeast, human, and plant pathogenic fungi (Table 2). For example, 16 *UBP* genes identified in *S. cerevisae* were found to be important for growth, nutrient utilization, stress response, energy metabolism, and sexual reproduction [43,44,45]. Disruption of *S. cerevisiae DOA4*, *UBP6*, *UBP8*, *UBP10,* and *UBP14* have all resulted in increased ubiquitin levels [46]. More specifically, DOA4 and UBP10 are required for carbon utilization and nitrogen utilization, respectively [44]; UBP14 is required for adaptation to the presence of glucose to mediate the degradation of enzymes involved in gluconeogenesis [47]; and UBP15 plays key roles in stress response [48].

In *C. neoformans*, all 19 *DUB* genes are not essential, whose mutants have been described and have shown normal growth under standard conditions [54,59]. According to a phenotypic analysis, it was found that DUBs, UBP5, DOA4, UBP13, and UBP14 were required for pigmentation, and UBP5 could be the major DUB required for stress response with roles in virulence, capsule formation, and sporulation [54]. In *M. oryzae*, all of the *UBP* genes have been systematically analyzed [49].

In *M. oryzae*, 11 putative *UBP* genes have been characterized (Table 2) [49]. The results have shown that *UBP* genes play various roles in hyphal growth, conidium formation, stress response, nutrient utilization, and infection-related structure formation [49]. Evaluation of the expression profiles of these *UBP* genes has demonstrated that *UBP3*, *UBP6*, *UBP1,2* and *UBP14* are constantly expressed at high levels during all tested developmental stages, while *UBP1*, *UBP13,* and *UBP15* are expressed at low levels. Four genes in mycelia, seven genes in conidia and appressoria, and five genes in invasive hyphae have high expression levels [49]. These data suggest that *UBP* genes play various biological functions during different development and infection stages. Among these, *M. oryzae UBP* genes, i.e., *UBP1*, *UBP3*, *UBP4*, *UBP8,* and *UBP14* are required for colony growth, nearly all *UBP* genes are required for conidia production, and most of them are required for full virulence (except for *UBP2* and *UBP16*) [49]. Accordingly, except for *UBP2* and *UBP16*, all other UBP genes are required for oxidative stress response, but they are differently required for responses to other stresses [49]. Detailed phenotypic roles of these DUBs in human and plant pathogenic fungi are discussed below.

### 3.2. DUBs in Fungal Growth

For fungi, hyphal growth is relevant to cell division, cell cycle, nutrient metabolism, and energy metabolism. In *S. cerevisiae*, deubiquitination is an important process to regulate energy uptake and the cell cycle for growth [60]. In human pathogen *C. neoformans*, *UBP5* deletion mutant is temperature sensitive, it grows slower at 37 °C and loses growth ability at 39 °C [54]. The mutant still grows slower at 30 °C in a rich medium. This phenotype may be due to the functions of *UBP5* in nutritional utilization and cell cycle regulation [54]. Similarly, the orthologs of *CnUBP5* in *S. cerevisiae* (*UBP15*) and in *C. gattii* (*CgUBP5*) are also closely relevant to their proliferation or propagation [54].

In *M. oryzae*, *UBP1*, *UBP3*, *UBP4*, *UBP8,* and *UBP14* deletions have all been reported to lead to defects in vegetative growth (Table 2) [49]. The growth defect of Δ*MoUBP14* was much more severe than other *UBP* mutants. The Δ*MoUBP14* mutant colony was much whiter, suggesting a regulatory role of UBP14 in mycelial melanin-related pigmentation [49,53]. The cell cycle of Δ*MoUBP14* was affected, demonstrated by a shorter hyphal tip cell [53]. Another reason why Δ*MoUBP14* grows slower is that *UBP14* is involved in nutrient utilization. Similar phenotypes in growth have also been found in the Δ*MoUBP3* mutant, with an evident cell cycle defect in the hyphal tip cell [50]. The Δ*MoUBP4* mutants have shown reduced colony diameter and compact mycelium mass when incubated in liquid complete medium (CM) [51]. The Δ*MoUBP8* mutant is slightly reduced in both mycelial growth and melanin synthesis [52]. In *S. scitamineum*, the DUB gene *SsCI33130* is not relevant to growth [57].

### 3.3. DUBs in Fungal Spore Production

Many fungi can produce asexual spores, called conidia, which germinate to form mycelium in water and adequate nutrition conditions, or to form infection structures on host surfaces. The formation of asexual spores is very important for the life cycle of plant pathogenic fungi, and conidiogenesis is a key step in the infection process. In the plant pathogenic fungus *M. oryzae*, most of the identified *UBP* genes (except *UBP2* and *UBP15*) have been found to be required for conidiogenesis [49]. The *UBP1*, *UBP3*, *UBP4*, *UBP6*, *UBP8*, *UBP12*, *UBP13*, *UBP14,* and *UBP16* deletion mutants are evidently decreased in conidiation. Only a few visible spores could be found on culture plates of the Δ*UBP8* and Δ*UBP14* mutants. Accordingly, conidiophore formation of these *UBP* mutants was also much more sparse as compared with the wild type.

In *A. alternata*, the AaCSN5 gene has been reported to be required for conidiation. The Δ*AaCSN5* mutant is severely reduced in conidia formation [58]. In another plant pathogenic fungus *Pestalotiopsis fici* [61], and in non-plant pathogenic fungus *N. crassa*, deletion of *CSN5* homolog genes also severely affects conidia production [62,63]. Interestingly, the homologous gene of *CSN5* in *A. nidulans* seems not to be involved in asexual spore production, but is required for the sexual developmental cycle [64].

### 3.4. DUBs in Fungal Sexual Reproduction

Sexual reproduction of fungi, similar to that of other organisms, includes a combination of two compatible nuclei. Through this method, fungi can create a diverse genetic background, which is conducive to their adaptation to the environment and host. It has been reported that in the human pathogen *C. neoformans*, UBP5 is essential for sexual reproduction and helps to produce the main infectious propagules (basidiospores) of human *cryptococcosis* [54]. The mating between WT and the *UBP5*Δ mutant cannot produce any filaments, due to blockage at the cell fusion point. This phenotype of *UBP5*Δ may be partially caused by blocking MFa pheromone gene expression [54].

For the plant pathogenic fungus *S. scitamineum*, sexual mating is closely related to its pathogenicity. The OTU1 deubiquitinase gene *SsCI33130* regulates the expression of genes involved in the synthesis of cAMP and tryptophol [57]. The cAMP and tryptophol are small molecule signaling substances required for the sexual mating of *S. scitamineum*. CSN5 in *A. nidulans* also seems to be required for the sexual developmental cycle [64].

### 3.5. DUBs in Fungal Stress Response

Various extracellular or intracellular stresses, such as high temperature, oxidative damage, high salt, and antifungal drugs, lead to the accumulation of misfolded or damaged proteins and cause cell damage. Removal of these misfolded or damaged proteins through ubiquitin-dependent proteolysis can prevent cell damage. Ubiquitination is an essential element during stress response, which is required for resistance to heat stress, oxidative stress, and other stresses [2,3]. Conversely, deubiquitination mediated by DUBs is required for maintaining ubiquitin homeostasis through processing of the ubiquitin precursors or proofreading of the ubiquitin-conjugated proteins [2,3,6], and therefore, is also important for stress response in fungi. Ubiquitination and deubiquitination may be important modification mechanisms of fungal stress response-related signaling pathways.

In *C. neoformans*, many genes encoding E2 are significantly upregulated in the HOG pathway mutants. In addition, some genes encoding components of Ca2+/calcineurin signaling and MAPK signaling pathways also change expression in the deletion mutant of CnUBP5 [54]. UBP5 could be the major DUB to regulate resistance to stresses in *C. neoformans*. During infection, *C. neoformans* needs to encounter temperature stress of the host cells, as well as other stresses including oxidative and nitrosative stress, salt stress, and antifungal agents. Interestingly, the *UBP5*Δ mutant is sensitive to all the abovementioned stresses, as well as cell wall and cell membrane perturbing agents [54], reflecting a major role of CnUBP5 in stress adaptation. CnUBP5 may coordinate with UBI4 to keep a balance of cellular ubiquitin under stresses. In addition to CnUBP5, *C. neoformans* DOA4 is also required for response to stresses of high sodium ions and cell wall perturbing agents SDS and caffeine [54]. CnDOA4 and CnUBP13 are also required for responses to antifungal drugs such as fluconazole and caspofungin [54].

In *C. gattii*, the DUB UBP5 also plays a key role in stress response. The deletion mutant of *UBP5* is sensitive to various stressors, including high temperature stress, oxidative stress, NO stress, salt stress, antifungal drugs, etc. [55]. It is predicted that *Cryptococcus* spp. UBP5 could regulate substrates in different stress responsive signaling pathways to encounter various cellular and environmental stresses.

In *M. oryzae*, most of the *UBP* genes are responsive to different stresses, positively or negatively [49]. For instance, positive UBP regulators include UBP1, UBP3, UBP8, UBP12, and UBP14, while UBP1, UBP2, and UBP4 are negative UBP regulators in oxidative stress response, and UBP2 and UBP4 are negative UBP regulators in cell wall stress response, among which, MoUBP14 could be a main regulator in stress response. The Δ*UBP14* mutant is more sensitive to various stresses, such as oxidative stress, osmotic stress, salt stress, cell wall perturbing agents, alkaline pH stress, as well as high temperature stress [49,53].

All of the above studies suggest that DUBs, especially UBPs, are very important for stress adaptation in human and plant pathogenic fungi, which can help fungi to encounter harsh host and environmental conditions for survival and infection.

### 3.6. DUBs in Fungal Nutrient Utilization

In fungi, nutrients provide energy and substrates for biosynthesis and catabolism, as well as play important roles in cellular regulation. Whether *UBP* genes are required for carbon source utilization has been evaluated in *M. oryzae*. On minimal medium (MM) plates supplemented with each of glucose, NaAc, ethanol, and glycerol as the sole carbon sources, growth of the Δ*UBP4* and Δ*UBP14* mutants were significantly reduced [49,51,53]. This result suggests that *UBP4* and *UBP14* are required for usage of all the tested carbon sources, therefore, acting as positive regulators of carbon source utilization. On the contrary, *UBP1* and *UBP8* function as negative regulators of carbon source utilization [49], demonstrating a complex regulatory mechanism in plant pathogenic fungi. In another plant pathogenic fungus *A. alternata*, AaCSN5 plays a role in nitrogen metabolism, and several amino acid metabolism pathways are positively regulated by it [58].

### 3.7. DUBs in Fungal Virulence

Many filamentous fungi are pathogenic to humans or plants. Diseases caused by pathogenic fungi threaten both plant health and human health.

The human pathogenic fungus *C. neoformans* arranges multiple pathogenic factors to overcome host cell stresses and to cause infection of the host. Extracellular polysaccharide capsule, melanin, and hydrolytic enzyme are key pathogenic factors in *C. neoformans*, which are essential for pathogenicity [65]. *CnUBP5* deletion leads to a defect in capsule attachment. UBP5 probably affects the capsule by involvement in cell wall polysaccharide assembly but not in the capsule synthesis or secretion [54]. Another important virulence-related DUB in *C. neoformans* is DOA4, whose deletion mutant is dramatically attenuated in virulence in the macrophage assay [54]. Thermotolerance in host cells is an important virulence factor that is regulated by both UBP5 and DOA4 [54].

In another human pathogenic fungus *C. gattii*, the *CgUBP5*Δ mutant is significantly attenuated in virulence in a mammalian host [55]. Interestingly, this mutant shows a slightly enhanced capsule and melanin production, which is different to that of the *CnUBP5*Δ mutant. Thermotolerance is also reduced in the *CgUBP5*Δ mutant [55], which is part of the factor that leads to the reduced survival rate of mutants in macrophages. Therefore, CgUBP5 is necessary for the survival of *C. gattii* in the lung space and its extrapulmonary transmission.

For plant pathogenic fungi, their pathogenicity is closely associated with infection-related morphogenesis and form special structures for host infection [66]. The rice blast fungus *M. oryzae* forms a special structure called appressorium, which forms a penetrating peg and penetrates the host plant epidermis through the physical force provided by the swelling pressure in the appressorium [66]. Whether each *UBP* gene in *M. oryzae* is involved in regulating virulence and infection processes has been evaluated [49]. The results have demonstrated that *UBP1*, *UBP3*, *UBP4*, *UBP6*, *UBP8,* and *UBP14* are relevant to the virulence of *M. oryzae*, while *UBP2* and *UBP16* are not [49]. The deletion mutants of those virulence-related genes are normal in appressorium formation, however, Δ*UBP14* was severely reduced in appressorium-mediated penetration. The Δ*UBP1*, Δ*UBP3*, Δ*UBP4*, Δ*UBP6*, and Δ*UBp8* mutants, as well as Δ*UBP14*, are normal in penetration, but are blocked in infection hyphae formation and extension [49]. All of the virulence-related genes are required for eliminating ROS accumulation of the host plant cells, suggesting that these *UBP* genes are important for host cellular oxidative stress resistance, as one of the roles in regulating invasive growth.

The Δ*UBP14* mutant totally loses its virulence due to several reasons. First, the Δ*UBP14* mutant is blocked in appressorium-mediated penetration, which is due to poor utilization of glycogen and lipid storage, defect in cell wall integrity, and a reduction in appressorial turgor [53]. Second, the mutant is also retarded in invasive growth, which is partly attributed to the sensitivity to distinct stresses (including oxidative stress), and a reduction in nutrient utilization [53]. The severe reduction of virulence in the Δ*UBP8* mutant is synthetically attributed to defects in melanin synthesis, appressorium turgor pressure, and ROS eliminating, as well as adaptation to other stresses such as salt stress, osmotic stress, and cell wall perturbing stresses [52]. Expressions of two pigment synthesis genes (*MoBUF1* and *MoRSY1*) are significantly reduced in the Δ*UBP8* mutant [52], suggesting a regulatory mechanism of *UBP8* in melanin synthesis. The Δ*UBP4* mutant is delayed in conidial glycogen and lipid droplets utilization, but forms normal appressorium turgor [51]. Therefore, the reduced virulence in the Δ*UBP4* mutant could mainly be due to defect in stress response.

*MoATG4* deletion, a gene encoding protease of Ubl Atg8 in *M. oryzae*, resulted in reduction of appressorium formation and maturation [67]. The ∆*MoATG4* mutant accumulates lower appressorium turgor pressure and fails to penetrate rice and barley [67].

In plant pathogenic fungus *A. alternata*, deletion of DUB gene *AaCSN5* significantly reduces its virulence and necrotic lesions on citrus leaves [58]. The reduction of the virulence is due to activation of the host immune response and a decrease in toxin production [58]. However, *AaCSN5* is not relevant to oxidative stress tolerance and host ROS scavenging.

In *S. scitamineum*, DUB gene *SsCI33130* deletion also results in a severe reduction in the pathogenicity to host plants [57]. Interestingly, it seems that *SsCI33130* does not affect the fungal growth and development, stress response, and other phenotypes of *S. scitamineum*. The pathogenicity reduction of the deletion mutant is only attributed to sexual mating [57], which is the primary condition for dikaryotic mycelia formation and pathogenicity development in *S. scitamineum*.

## 4. Regulatory Mechanisms Mediated by DUBs in Different Pathogenic Fungi

DUBs are part of the ubiquitin–proteasome system, which are involved in some cellular key processes such as protein quality control, DNA damage repair, and cell cycle regulation, etc. [5]. Recent studies in pathogenic fungi have revealed some regulatory mechanisms mediated by DUBs, including appressorium associated signaling pathways, autophagy, nutrient utilization, histone modification, endocytosis, proteasome function, and circadian clock.

### 4.1. DUBs and Cellular Signaling Pathways

As a central nutrient signaling pathway, the target of rapamycin (TOR) signaling pathway negatively regulates macro-autophagic processes [68]. This signaling pathway works in parallel with the Ras/PKA signaling pathway to regulate appressorium formation of plant pathogenic fungi such as *M. oryzae* [69]. It has been reported that in *M. oryzae*, UBP3-mediated ribophagy may be regulated by the TOR signaling pathway [50]. The ∆*UBP3* mutant is blocked in ribophagy and is very sensitive to rapamycin. This phenotype is consistent with a study in *S. cerevisiae*, in which the TOR signaling pathway activated UBP3p-mediated ribophagy processes [70]. However, it is still unknown whether UBP3-mediated ribophagy plays important roles in appressorium formation and maturation.

**Figure 3:**
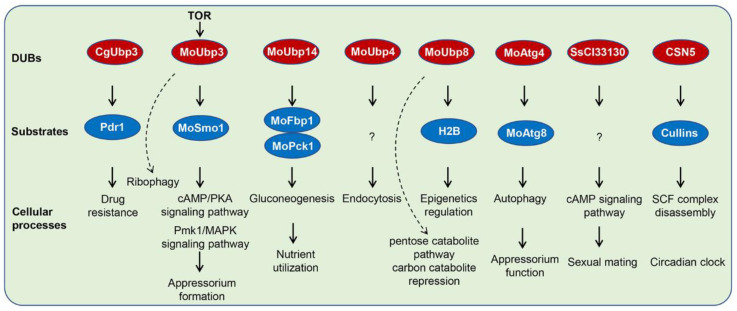
Regulatory mechanisms of DUBs in pathogenic fungi.

In general, the TOR signaling pathway is antagonistic with the Ras/PKA signaling pathway, and increased sensitivity to rapamycin usually leads to overactivation of the Ras/PKA signaling pathway [68,69]. Consistent with this, UBP3 deletion has resulted in overactivation of the RAS2/PKA signaling pathway in *M. oryzae* [50], which was important for appressorium formation. This regulatory mechanism is achieved by UBP3-mediated deubiquitination of Smo1, which is a GTPase-activating protein (RasGAP) involved in regulating the balance of Ras proteins in *M. oryzae* [71]. Moreover, UBP3-mediated deubiquitination of Smo1 also regulates the Pmk1 MAPK signaling pathway, another main signaling pathway regulating appressorium formation. As a result, the Δ*UBP3* mutant is delayed in appressorium formation and maturation, fails to form enough appressorial turgor for penetration, and is reduced in virulence [50]. Therefore, UBP3-mediated deubiquitination of Smo1 is a molecular switch for Ras signaling. During appressodium formation, UBP3 expresses at a low level to promote ubiquitination of Smo1, then increases the protein level of GTP-bound active RAS2. Subsequently, the cAMP-PKA and Pmk1-MAPK signaling pathways are activated to promote appressorium formation (Figure 3) [50].

In *S. scitamineum*, another DUB gene *SsCI33130* has also been found to be involved in the cAMP signaling pathway. *SsCI33130* deletion leads to a defect in sexual mating in *S. scitamineum*, while exogenous addition of cAMP restores the sexual mating of the mutant [57]. Expression of two cAMP synthesis-related genes, *ARO8* and *UAC1*, are regulated by SsCI33130, suggesting a potential regulatory mechanism of the cAMP signaling pathway [57].

### 4.2. DUBs and Autophagy

Autophagy plays a key role in infection of the plant pathogenic fungi, for example, in appressorium formation of the rice blast fungus *M. oryzae*. There are around 20 *ATG* genes related to the autophagy process in *M. oryzae*, among which, Atg8 and Atg12 are UbLs [72]. The DUB of Atg8 is Atg4 as reported in *S. cerevisiae* [73]. *M. oryzae* Atg4 can complement ScAtg4 in yeast, and interact with MoAtg8 and cleave it in vitro [67]. The *MoATG4* gene is induced by starvation and controls autophagic cell death during appressorium formation (Figure 3) [67].

Under nitrogen starvation, ribosomes are rapidly degraded in *S. cerevisiae*, which is mediated by a selective autophagy process, so called ribophagy [74]. A similar process is also found in *M. oryzae* (Figure 3) [50]. The ribosome marker protein Rpl25 is degraded during the ribophagy process, which is UBP3 dependent and induced by rapamycin treatment and nitrogen starvation conditions [50]. In the yeast *S. cerevisiae*, full function of UBP3p requires BRE5p to form a complex. Studies have also found that the UBP3/BRE5 complex not only regulated ribophagy, but was also related to the regulation of mitophagy [69]. The detailed mechanism and function of the UBP3/BRE5 complex-mediated autophagic processes in pathogenic fungi requires further study.

### 4.3. DUBs and Fungal Carbon Source Utilization

Nutrient utilization is essential for fungal development and formation of infection-associated structures, such as appressorium and invasive hyphae in plant pathogenic fungi. We have mentioned above that *M. oryzae* UBP14 regulates carbon source utilization. This regulation is at least achieved by UBP14-mediated deubiquitination of FBP1 and PCK1 (Figure 3). FBP1 and PCK1 are two key enzymes that catalyze gluconeogenesis. After the addition of glucose, FBP1 and PCK1 are subjected to protein degradation, by which they are rapidly degraded through the ubiquitin proteasome system. When glucose was supplied to *M. oryzae* growing in a low glucose medium, FBP1 and PCK1 were degraded in a short time (∼12 h) [53]. Therefore, UPS-mediated degradation of FBP1 and PCK1 may be related to coordinating gluconeogenesis and glycolysis balance, thereby facilitating the fungi to utilize nutrients during infection.

MoUBP8 is also involved in carbon utilization, through a regulatory mechanism different from MoUBP14 [52]. MoUBP8 is mainly associated with the pentose catabolite pathway and carbon catabolite repression (CCR) (Figure 3). In the pentose catabolic pathway, D-xylose is converted to xylitol and D-xylulose-5-phosphate for further utilization, however, utilization of D-xylose is blocked in the Δ*MoUBP8* mutant. In addition, the Δ*MoUBP8* mutant also shows a defect in CCR repression, demonstrated by more sensitivity to AA but resistance to 2-DG [52].

### 4.4. DUBs and Histone Modification

In *S. cerevisiae*, the transcriptional activation process can be regulated by UBP8-mediated deubiquitination [75]. The UBP8p is a component of the DUB module, which is composed of UBP8, SGF11, SUS1, and SGF73 [76]. The UBP8 DUB module is responsible for H2B deubiquitination, which coordinates with the histone SAGA complex (Spt-Ada-Gcn5-acetyltransferase complex) to activate gene expression in *Candida albicans* (Figure 3) [76,77]. Another DUB, UBP10, is also required for deubiquitination of H2B in yeast [76], however, the downstream regulatory mechanism, either UBP8 or UBP10, has not been revealed in the pathogenic fungi and needs to be uncovered in the future.

### 4.5. DUBs and Endocytosis

DUBs participate in the endocytic pathway and are also involved in other intracellular traffic types. In *S. cerevisiae*, the DUB DOA4/UBP4 plays a role in recycling ubiquitin at late endosome. Disruption of *S. cerevisiae DOA4* results in defects of many ubiquitin-related processes [78]. The endosomal sorting complex required for transport III (ESCRT-III) proteins BRO1 and SNF7 can activate the deubiquitinating activity of DOA4 by recruiting it to the endosomes [79]. Although a yeast hybrid assay has confirmed the interaction between MoUBP4 and MoSNF7/MoBRO1 in *M. oryzae* [51], the biological functions and regulatory mechanisms of the MoUBP4-MoBRO1-MoSNF7 module are still unknown and require exploration.

### 4.6. DUBs and Circadian Clock

The DUB protein CSN5 is one component of the COP9 signalosome (CSN), which contains eight subunits and functions as a deneddylation enzyme to remove NEDD8 from tagged proteins [16]. The cullin subunit of the SCF-FWD1 complex is tagged with a NEDD8 molecule while it is part of the SCF complex [16]. NEDD8 can tag to the cullin subunit of the SCF-FWD1 complex to be part of the SCF complex as a ubiquitin E3 ligase [16]. CSN5-mediated cullin deneddylation can regulate SCF complex disassembly, which is required for FRQ ubiquitination [62,63]. In the CSN mutants, FRQ is accumulated and rhythmicity is lost [62,63]. However, the CSN5-mediated regulatory mechanism in circadian clock requires more attention.

### 4.7. DUBs and Drug Resistance

Drug resistance is very important for adaptation and survival of human and plant pathogenic fungi. In *C. glabrata*, BRE5 and UBP3-mediated deubiquitination of PDR1 play key roles in drug resistance [56]. UBP3 is co-purified with BRE5 and PDR1 [56]. The deubiquitinating enzyme UBP3 has been found to function together with BRE5 for its activity in *S. cerevisiae* [80]. This UBP3:BRE5 deubiquitinase complex deubiquitinates the drug resistance transcription factor PDR1, and negatively regulates its activity to regulate drug resistance (Figure 3).

## 5. Conclusions

The past 10 years have yielded much new knowledge regarding DUBs in human and plant pathogenic fungi. Here, we demonstrated the physiological roles of DUBs in fungal growth, development, asexual spore reproduction, sexual mating, stress response, nutrient utilization, and infection processes. These studies have emphasized key roles of DUBs in virulence of both human and plant pathogenic fungi, especially in the human fungal pathogen *C. neoformans* and the plant fungal pathogen *M. oryzae*. In this article, we have primarily discussed the mechanisms of the regulatory roles of DUBs in cellular signaling pathways, autophagy, carbon source utilization, histone modification, endocytosis, and circadian clock.

As is evident from the brevity of this review, the potential roles of deubiquitinases in human and plant pathogenic fungi remains largely unexplored. Much work also remains to be done with the DUBs in regulating pathogenicity, in addition to the human fungal pathogen *C. neoformans* and the plant fungal pathogen *M. oryzae*. The substrates of the enzyme and the molecular mechanisms of DUBs in virulence remain largely unknown. Future investigations will focus on high-throughput identification of each DUB targets by ubiquitination proteomics, which would lead to a comprehensive understanding of the regulatory mechanism. Investigation of the upstream regulation of DUBs, DUB coordination, and interaction with other pathways or complexes are also required. In addition, the regulatory mechanisms of DUBs in transcription, translation, epigenetics, and post-translational modifications would be of interest.

At present, small molecule inhibitors have been identified to suppress DUB targets to control cancers and virus infection in humans [81,82]. Considering that DUBs play key roles in the pathogenicity of fungal pathogens, it is possible to identify potential antimicrobial drugs or fungicides in the future.

## Figures and Tables

**Table 1:** DUBs in Saccharomyces cerevisiae, Cryptococcus neoformans, and *Magnaporthe oryzae*.

	*S. cerevisiae*	*C. neoformans*	*M. oryzae*
UBPs	UBP1	-	MGG_04031
	UBP2	CNAG_07333	MGG_00877
	UBP3	CNAG_06920	MGG_05996
	UBP4	CNAG_00757	MGG_04957
	UBP5		-
	UBP6	CNAG_03109	MGG_02970
	UBP7	-	-
	UBP8	CNAG_06637	MGG_03527
	UBP9	CNAG_02395	-
	UBP10	CNAG_00455	-
	UBP11	-	-
	UBP12	CNAG_07717	MGG_05666
	UBP13	CNAG_02395	MGG_09907
	UBP14	CNAG_05708	MGG_08270
	UBP15	CNAG_05650	MGG_07468
	UBP16	-	MGG_04494
UCHs	YUH1	CNAG_00180	MGG_06319
			MGG_01683
OTUs	OTU1	-	MGG_03532
	OTU2	CNAG_02004	MGG_11505
MINDY	MIY1	-	MGG_03906
	YGI2	-	MGG_05274
JAMM	RPN11	CNAG_07028	MGG_16706
		CNAG_04809	MGG_05274
		CNAG_06563	MGG_01059
			MGG_10653

**Table 2:** Fungal DUBs and their functions.

Fungal DUBs	Biological Functions	Mechanisms	Ref.
*Magnaporthe oryzae* Ubp1	Growth, conidiation, virulence, stress response		[49]
*M. oryzae* UBP2	No evident function was found		[49]
*M. oryzae* UBP3	Growth, conidiation, virulence, stress response	Regulate ribophagy and GTPase-activating protein Smo1, cAMP, and Pmk1-MAPK signaling	[49,50]
*M. oryzae* UBP4	Growth, conidiation, virulence, stress response, nutrient utilization	Recycle Ub at the late endosome, endocytosis	[49,51]
*M. oryzae* UBP6	Conidiation, virulence, stress response		[49]
*M. oryzae* UBP8	Growth, conidiation, virulence, stress response	Component of SAGA complex, regulates transcriptional activation by deubiquitinating H2B	[49,52]
*M. oryzae* UBP12	Growth, conidiation, virulence, stress response		[49]
*M. oryzae* UBP13	Conidiation, virulence, stress response		[49]
*M. oryzae* UBP14	growth, conidiation, virulence, stress response, nutrient utilization	Regulate gluconeogenesis key enzymes MoFBP1 and MoPCK1	[49,53]
*M. oryzae* UBP15	Virulence, stress response		[49]
*M. oryzae* UBP16	Conidiation		[49]
*Cryptococcus neoformans* DOA4	Sporulation, pigment production, capsule formation, virulence, stress response, sexual reproduction		[54]
*C. neoformans* UBP13	Stress response, pigment production		[54]
*C. neoformans* UBP14	Stress response, pigment production		[54]
*C. neoformans* UBP5	Growth, sporulation, melanization, capsule formation, virulence, stress response, sexual reproduction	Major deubiquitinating enzyme for stress response	[54]
*Candida gattii* UBP5	Growth, stress response, virulence		[55]
*Candida glabrata* BRE5	Azole resistance	Association with UBP3, negatively regulate transcription of *PDR1*	[56]
*Sporisorium scitamineum* SsCI33130	Sexual mating and pathogenicity	Regulate the synthesis of the small-molecule signaling substances (cAMP or tryptophol)	[57]
*Alternaria alternata* CSN5	Light response, sexual development, virulence, and secondary metabolism	Components of COP9 signalosome, regulate activity of cullin–RING ubiquitin E3 ligases	[58]

## Data Availability

Not applicable.
